# 胰岛素样生长因子受体基因单核苷酸多态性与晚期非小细胞肺癌铂类化疗疗效及预后的研究

**DOI:** 10.3779/j.issn.1009-3419.2012.02.01

**Published:** 2012-02-20

**Authors:** 愉生 陈, 慧 邵, 鸿茹 李, 丽丽 韩, 祥娥 张

**Affiliations:** 1 350001 福州，福建医科大学省立临床医学院 Fujian Provincial Medical College, Fujian Medical University, Fuzhou 350001, China; 2 350001 福州，福建省立医院心血管重点实验室 Key Laboratory of Cardiovascular Disease, Fujian Provincial Hospital, Fuzhou 350001, China

**Keywords:** 肺肿瘤, 化疗, 胰岛素样生长因子受体, 核苷酸多态性, Lung neoplasms, Chemotherapy, Somatomedin receptors, Single nucleotide polymorphism

## Abstract

**背景与目的:**

胰岛素样生长因子1受体（insulin-like growth factor 1 receptor, *IGF*-*1R*）基因是调节细胞生长分化的重要基因，胰岛素样生长因子2受体（insulin-like growth factor 2 receptor, *IGF*-*2R*）基因是潜在的肿瘤抑制基因，本研究旨在探讨胰岛素样生长因子受体基因IGF-1R+1013（G/A）、IGF-2R+1619（G/A）单核苷酸多态性与非小细胞肺癌（non-small cell lung cancer, NSCLC）患者以铂类药物为基础化疗方案的疗效及预后的关系。

**方法:**

经病理确诊的132例初治肺癌晚期患者经含铂方案化疗4周期后评价临床疗效，采用聚合酶链反应-限制性片段长度多态性检测IGF-1R+1013（G/A）和IGF-2R+1619（G/A）的基因型，分析其基因多态性与化疗临床受益率及生存期的关系。

**结果:**

未发现IGF-1R+1013（G/A）基因多态性和IGF-2R+1619（G/A）基因多态性与化疗疗效有明显关系（*P*＞0.05），联合分析未发现两基因多态性与化疗疗效有联合作用（*P*=0.975）。IGF-1R+1013（G/A）变异等位基因A携带者（GA+AA）的中位生存时间（middle survival time, MST）短于GG基因型携带者（*P*=0.017），IGF-2R+1619（G/A）变异等位基因A携带者（GA+AA）和GG基因型携带者的MST的差异无统计学意义（*P*=0.575）。联合分析发现两基因多态之间存在联合作用，同时携带IGF-1R+1013（G/A）突变等位基因A和IGF-2R+1619（G/A）突变等位基因A的患者（GA+AA）的MST为12个月，明显短于携带其它基因型的患者（*P*＜0.05）。*Cox*比例风险模型分析，IGF-1R+1013（G/A）基因多态性是影响NSCLC预后的独立因素（*P*=0.020），IGF-1R+1013（G/A）和IGF-2R+1619（G/A）联合基因多态性也是影响NSCLC预后的独立因素（*P*=0.025）。

**结论:**

IGF-1R+1013（G/A）基因多态性单独或联合IGF-2R+1619（G/A）基因多态性与晚期NSCLC生存期有关，可以在一定程度上判断晚期NSCLC患者的预后。

## Abstract (en)

**Background and objective:**

It has been proven that the insulin-like growth factor 1 receptor (*IGF*-*1R*) gene is an important regulator of many aspects of growth, differentiation, and development. The insulin-like growth factor 2 receptor (*IGF*-*2R*) gene is a negative mediator for carcinogenesis. The aim of this study is to investigate the relationship of IGF-1R+1013(G/A) and IGF-2R+1619(G/A) single nucleotide polymorphism (SNP) with platinum-based chemotherapy outcomes in advanced non-small cell lung cancer (NSCLC).

**Methods:**

A total of 132 patients with NSCLC were routinely treated with platinum-based chemotherapy, and their clinical responses were evaluated after four cycles of chemotherapy. IGF-1R+1013(G/A) and IGF-2R+1619(G/A) were genotyped using polymerase chain reaction-restrictive fragment length polymorphism. The relationship between IGF-1R+1013(G/A) and IGF-2R+1619(G/A) genotypes and the clinical benefit rate, as well as the median survival time (MST), was analyzed.

**Results:**

No significant association was found between IGF-1R+1013(G/A) and IGF-2R+1619(G/A) polymorphisms with clinical benefit (*P* > 0.05). Further, we found that the two SNPs could not work together (*P*=0.975). The MST of patients with IGF-1R+1013(G/A) genotypes with A allele (GA+AA) was significantly shorter than that of GG genotype carriers (*P*=0.017). There was no significant difference in MST in patients with IGF-2R+1619(G/A) A allele (GA+AA) carrier and GG genotype carrier (*P*=0.575). The two SNPs showed a synergistic effect on MST. Patients who carried a mutant allele A of IGF-1R+1013(G/A) and a mutant allele A of IGF-2R+1619(G/A) had a MST of 12 months, which was significantly shorter than that of patients with other genotypes (*P* < 0.05). Estimation by the *Cox* proportional hazards model showed that IGF-1R+1013(G/A) polymorphism is an independent prognostic factor (*P*=0.020), and IGF-1R+1013(G/A) polymorphism in combination with IGF-2R +1619(G/A) polymorphism is an independent prognostic factor in advanced NSCLC (*P*=0.025).

**Conclusion:**

IGF-1R+1013(G/A) polymorphism alone or in combination with IGF-2R +1619(G/A) polymorphism was associated with the overall survival period in patients with advanced NSCLC after treatment with platin-based chemotherapy, which might be a prognostic factor in platin-treated patients with advanced NSCLC.

非小细胞肺癌（non-small cell lung cancer, NSCLC）是发病率和死亡率最高的恶性肿瘤之一，80%确诊的NSCLC患者属于晚期，需进行以内科治疗为主的综合治疗。目前铂类药物联合吉西他滨、紫杉烷类或长春碱类药物治疗是主要标准方案，但总有效率仅为30%-40%^[1]^，个体遗传素质的不同可以导致化疗效果的差异。有研究^[2]^表明基因多态性是决定治疗反应性差异的重要因素。胰岛素样生长因子1受体（insulin-like growth factor 1 receptor, IGF-1R）和胰岛素样生长因子2受体（insulin-like growth factor 2 receptor, IGF-2R）作为胰岛素样生长因子信号途径的重要组成部分对调节细胞增殖、分化、凋亡有重要作用。IGF-1R与配体结合后激活酪氨酸激酶，促进细胞存活及抗细胞凋亡，但IGF-2R没有酪氨酸激酶活性，不能传导任何信号，是近年来备受关注的潜在肿瘤抑制因子。*IGF*-*1R*和*IGF*-*2R*基因中存在高度多态性，且某些碱基的突变与肿瘤的易感性有关，*IGF*-*1R*基因1013密码子鸟嘌呤（G）→腺嘌呤（A）突变与2型糖尿病患者的食管癌发生密切相关^[3]^，IGF-2R+1619（G/A）野生纯合型GG和*IGF*-*2*基因+3580位点AA纯合突变的组合对肝癌发生有保护作用^[4]^。在肿瘤化疗方面的研究^[5]^发现，体外抑制IGF-1R表达后顺铂作用肺癌细胞所引起的凋亡比例明显增加，然而其基因多态性与肺癌化疗疗效的研究未见报道。本研究旨在通过PCR-RFLP方法检测132例经铂类化疗的晚期肺癌患者IGF-1R+1013（G/A）和IGF-2R+1619（G/A）的基因型分布情况，探讨其基因多态性与化疗敏感性及生存期的关系，以期为临床化疗方案的选择提供依据。

## 1 材料与方法

### 1.1 研究对象

2009年10月-2011年10月期间住院的132例晚期NSCLC患者，纳入标准：①所有患者均经病理确诊并有可评估的病灶且无其它部位肿瘤；②均初次采用4周期的含铂类化疗方案，化疗前血常规、肝肾功能正常，心电图无明显异常，Karnofsky评分60分以上，预计生存期＞3个月；③所有病人均签署知情同意书且依从性好，能够进行随访。“吸烟”指从开始吸第1支烟到目前为止，1年内吸烟量≥100支或每周至少2支，连续1年以上。肿瘤按照UICC肺癌TNM分期第7版分期，组织类型根据2004年WHO公布的“肺及胸膜肿瘤组织学分类修订方案”将大细胞肺癌和未分化及未定性肺癌统称为其他型肺癌。

### 1.2 化疗方案及评价标准

根据肺癌的细胞类型和患者的全身情况制定化疗方案，132例患者接受以顺铂（cisplatin, DDP）或卡铂（carboplatin, CBP）为主的方案化疗，其中14例采用DDP/CBP联合伊立替康（CPT-11）治疗（IP方案），46例采用用DDP/CBP联合吉西他滨（gemcitabine, GEM）治疗（GP方案），28例采用采用DDP/CBP联合去甲长春碱（vinorelbine, NVB）治疗（NP方案），31例采用采用DDP/CBP联合紫杉醇（paclitaxel, TAX）或多西紫杉醇（docetaxel, DOC）治疗（TP方案），13例采用DDP联合培美曲塞二钠（pemet rexed disodium, PEM）治疗。上述各方案每3周为1个周期，疗程为4周期。根据2009年美国国立癌症研究所实体瘤客观疗效评定标准修订版RECIST 1.1进行疗效评价^[6]^，分为完全缓解（complete response, CR）、部分缓解（partial response, PR）、稳定（stable disease, SD）和进展（progressive disease, PD），设定CR+PR+SD且持续时间≥24周为临床受益。对化疗有效和病灶稳定的患者所采取的治疗策略应为停止化疗，进入观察随访期，出现新的病灶后再进行二线治疗或复治。生存时间指确诊Ⅲ期、Ⅳ期肺癌开始至死亡或末次随访时间，随访截止时间为2011年9月23日。所有患者均采用电话随访。

### 1.3 实验方法

#### 1.3.1 基因组DNA的提取

抽取每个研究对象外周肘静脉血5 mL，乙二胺四乙酸抗凝，离心并分离白细胞，用DNA提取试剂盒提取白细胞基因组DNA，核酸分光分度计对DNA的浓度和纯度进行检测，所有标本OD_260_/OD_280_值在1.8-2.0之间符合PCR要求，DNA置-20 ℃低温冰箱保存备用。

#### 1.3.2 聚合酶链反应-限制性片段长度多态性（PCR-RFLP）分析

根据文献设计引物，IGF-1R+1013（G /A）（rs2229765）的上游引物：5’-CAGGGGTCGTTTGGGATGGTC-3’；下游引物：5’-CCTGTGCTGC ATTTTGGCGGCTTTTC-3’。IGF-2R+1619（G/A）（rs629849）的上游引物：5’-AACAATGGTTAAAGCCGGATTG-3’，下游引物：5’-GGCCCGGGTGCAGCCAGGCACTG -3’。PCR扩增体系（25 μL）含0.4 μmol/L上下游引物，0.02 mmol/L dNTP，1.25 U TaKaRaTaq酶，1×PCR buffer，100 ng DNA模板，加蒸馏水至25 μL。IGF-1R PCR反应条件：94 ℃预变性10 min，94 ℃变性20 s，54 ℃退火20 s，72 ℃延伸30 s；共36个循环，末次循环后，72 ℃再延伸10 min，产物为207 bp。IGF-2R PCR反应条件：94 ℃预变性5 min，94 ℃变性30 s，67 ℃退火60 s，72 ℃延伸30 s；共30个循环，末次循环后，72 ℃再延伸15 min，产物为456 bp，以蒸馏水替代模板DNA作为阴性对照。取10 μL PCR反应产物、1 U限制性内切酶，10×buffer tango 2 μL，18 μL无菌去离子水，37 ℃水浴过夜。取5 μL酶切产物经3 g/L琼脂糖凝胶电泳，100 V、20 min，凝胶成像系统分型，IGF-1R PCR产物经酶切后出现103 bp、84 bp片段为GG纯合基因型，出现84 bp、123 bp片段为AA纯合基因型，出现103 bp、84 bp、123 bp片段为GA杂合基因型；*IGF*-*2R*基因PCR产物经酶切后出现307 bp和149 bp片段判定为GG纯合基因型，出现完整456 bp片段判定为AA纯合基因型，出现456 bp、307 bp和149 bp片段, 则判定为GA杂合基因型，结果见图 1和图 2。

**1:**
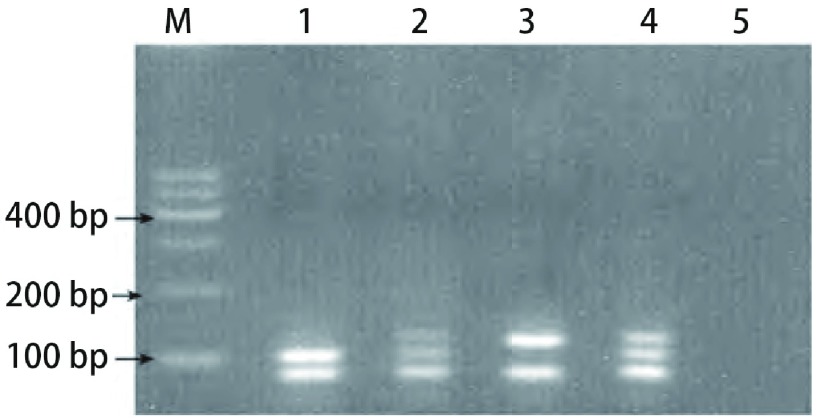
IGF-1R+1013（G/A）PCR产物经限制性内切酶*Mnl*I消化后的电泳图。M：marker；1：GG型；2，4：GA型；3：AA型；5：阴性对照。 The agarose gel electrophoresis of IGF-1R+1013(G/A) by restrict endonucleases *Mnl*I. M: maker; Lane1: GG genotype; Lane2, 4: GA genotype; Lane3: AA genotype; Lane5: negative control.

**2:**
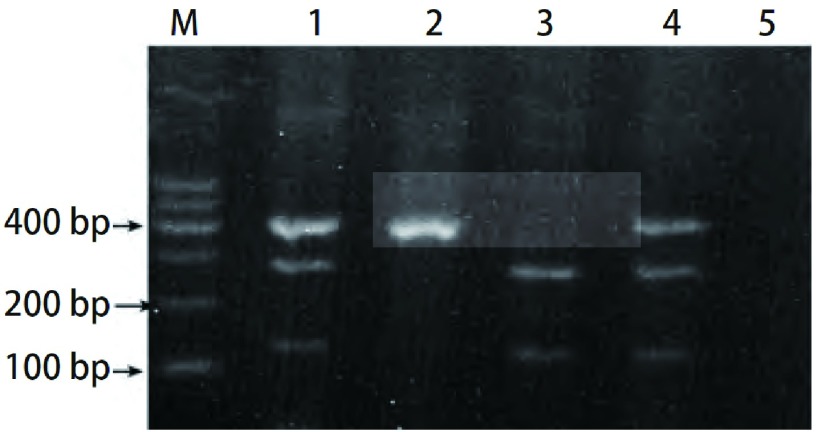
IGF-2R+1619（G/A）PCR产物经限制性内切酶*Mnl*I消化后的电泳图。M：marker；1，4：GA型；2：AA型；3：GG型；5：阴性对照。 The agarose gel electrophoresis of IGF-2R+1619(G/A) by restrict endonucleases *Nci*I. M: maker; Lane1, 4: GA genotype; Lane2: AA genotype; Lane3: GG genotype; Lane5: negative control.

#### 1.3.3 DNA测序

取50 μL PCR产物连同其上游引物由英潍捷基贸易有限公司采用双脱氧法在ABI_3730XL测序仪上进行单向测序，测序结果与PCR-RFLP方法一致。

### 1.4 统计学分析

采用SPSS 18.0统计软件，建立非条件*Logistic*回归模型评价*IGF*-*1R*和*IGF*-*2R*基因多态性与铂类化疗疗效的关联，以比值比（odd ratio, OR）及其95%可信区间（confidence in terval, CI）表示基因型与疗效的相关性。采用*Kaplan*-*Merier*法进行单因素生存分析，*Log*-*rank*检验比较不同基因型的生存期差异，*Cox*比例风险模型评估可能的因素对化疗疗效的影响。*P*＜0.05为差异有统计学意义。

## 2 结果

### 2.1 IGF-1R+1013（G/A）、IGF-2R+1619（G/A）基因多态性与铂类化疗疗效的关系

132例晚期肺癌患者中无CR，PR 38例，SD 62例，PD 32例，临床受益率75.6 %（100/132）。以性别、年龄、吸烟状况、组织类型、TNM分期、化疗方案、*IGF*-*1R*和*IGF*-*2R*的基因多态性为自变量，以化疗疗效为应变量做非条件*Logistic*回归分析，男性患者的临床受益率低于女性患者（*P*=0.023）；*IGF*-*1R*和*IGF*-*2R*各等位基因符合Hardy-Weinberg平衡定律，IGF-1R+1013（G/A）GG、GA、AA基因型携带者和变异等位基因A携带者（GA+AA）的临床受益率分别为75.5%、75.4%、78.6%和75.9%，差异无统计学意义（*P*＞0.05）；IGF-2R+1619（G/A）GG、GA、AA基因型携带者和变异等位基因A携带者（GA+AA）的临床受益率分别为75.3%、76.6%、75.0%和76.5%，差异无统计学意义（*P*＞0.05）。联合分析发现IGF-1R+1013（G/A）基因型与IGF-2R+1619（G/A）基因型对化疗疗效无协同作用（*P*=0.975）。见表 1。

**1:** 晚期肺癌患者临床特征、*IGF*-*1R*和*IGF*-*2R*的基因型与铂类化疗疗效的关系 The relationship among clinical features, *IGF*-*1R* and *IGF*-*2R* genotypes and efficacy of platinum- based chemotherapy

Characteristics	*n*	CR+PR+SD (%)^d^	PD (%)^d^	OR (95%CI)^e^	*P*^e^
Gender					
Female	47	39 (83.0)	8 (17.0)		
Male	85	61 (71.8)	24 (28.2)	0.25 (0.07-0.83)	0.023
Age (year)					
＜60	65	47 (72.3)	18 (27.7)		
≥60	67	53 (79.1)	14 (20.9)	2.15 (0.80-5.80)	0.129
Smoking status					
No smoking	65	47 (72.3)	18 (27.7)		
Smoking	67	53 (79.1)	14 (20.9)	3.23 (0.99-10.58)	0.053
Histology^a^					
SCC	33	29 (79.9)	4 (12.1)	1.00	-
AC	70	51 (72.9)	19 (27.1)	0.42 (0.11-1.55)	0.190
Others	29	20 (69.0)	9 (31.0)	0.39 (0.08-1.91)	0.340
pTNM stage					
Ⅲ	53	46 (86.8)	7 (13.2)		
Ⅳ	79	54 (68.4)	25 (31.6)	0.40 (0.14-1.10)	0.076
Chemotherapy regimen^b^					
IP	14	9 (64.3)	5 (35.7)	1.00	-
GP	46	34 (73.9)	12 (26.1)	1.11 (0.20-6.18)	0.905
NP	28	23 (82.1)	5 (17.9)	1.55 (0.24-9.91)	0.645
TP	31	24 (77.4)	7 (22.6)	1.47 (0.24-9.05)	0.676
DDP+PEM	13	10 (76.9)	3 (23.1)	1.37 (0.19-9.68)	0.753
IGF-1R					
GG	49	37 (75.5)	12 (24.5)	1.00	-
GA	69	52 (75.4)	17 (24.6)	0.85 (0.34-2.16)	0.739
AA	14	11 (78.6)	3 (21.4)	0.88 (0.18-4.34)	0.879
GA+AA^c^	83	63 (75.9)	20 (24.1)	0.86 (0.35-2.10)	0.737
IGF-2R					
GG	81	61 (75.3)	20 (24.7)	1.00	-
GA	47	36 (76.6)	11 (23.4)	1.23 (0.47-3.23)	0.672
AA	4	3 (75.0)	1 (25.0)	1.27 (0.10-16.46)	0.875
GA+AA^c^	51	39 (76.5)	12 (23.5)	1.24 (0.49-3.13)	0.656
^a^ SCC: squamous cell carcinoma; AC: adenocarcinoma; Others: include adenosquamocarcinoma, bronchioloalveolar carcinoma, large-cell carcinomas, unclassified non-small cell lung eancer. ^b^ IP: cisplatin/carboplatin+CPT-11; GP: cisplatin/carboplatin+gemcitabine; NP: cisplatin/carboplatin+vinorelbine; TP: cisplatin/carboplatin+paclitaxel/docetaxel; DDP+PEM: cisplatin+pemet rexed disodium.^c^ Genotypes with A allele (either the heterozygous GA or the homozygous AA genotypes); ^d^ CR:complete response; PR: partial response; SD:stable disease; PD: progression disease. ^e^ Adjusted for age, gender, smoking status, histology, clinical stage, chemotherapy regimen and IGF-1R and IGF-2R.					

### 2.2 IGF-1R+1013（G/A）、IGF-2R+1619（G/A）基因多态性与生存期的关系

至随访截止时132例晚期肺癌患者中55例死亡，无失访者，治疗后中位生存时间（middle survival time, MST）为18个月。IGF-1R+1013等位基因A携带者（GA+AA）的MST短于GG基因型携带者（*P*=0.017），IGF-2R+1619等位基因A携带者（GA+AA）与GG基因型携带者相比，MST差异无统计学意义（*P*=0.575）（表 2）。联合*IGF*-*1R*和*IGF*-*2R*基因型分析显示，两基因联合多态性与NSCLC的生存期有关，与同时携带这两个基因的GG基因型的个体相比，携带IGF-1R+1013（G/A）突变等位基因A（GA+AA）同时携带IGF-2R+1619（G/A）GG基因型的患者MST短（*P*=0.031）；同时携带IGF-1R+1013（G/A）突变等位基因A（GA+AA）和IGF-2R+1619（G/A）突变等位基因A（GA+AA）的患者的MST也短（*P*=0.041）（表 3，图 3）。

**2:** IGF-1R+1013（G/A）、IGF-2R+1619（G/A）基因型与肺癌生存期的关系 The relationship among polymorphisms of IGF-1R+1013(G/A)、IGF-2R+1619(G/A) and median survival time (MST)

Gene	*n* (%)	MST/month	95%CI	*χ*^2^	*P*
IGF-1R				5.721	0.017
GG	49 (37.1)	21	18.60-24.77		
GA+AA	83 (62.9)	14	9.78-18.22		
IGF-2R				0.314	0.575
GG	81 (61.4)	22	13.16-30.84		
GA+AA	51 (38.6)	16	11.41-20.60		

**3:** IGF-1R+1013（G/A）、IGF-2R+1619（G/A）联合基因型与肺癌生存期的关系 The relationship among combined polymorphisms of IGF-1R+1013(G/A)、IGF-2R+1619(G/A) and MST

IGF-1R+1013 (G/A)	IGF-2R+1619(G/A)	*n* (%)	MST (95%CI)	*P*
GG	GG	30 (22.7)	27 (18.74-29.27)	Reference
GG	GA+AA	19 (14.4)	22 (0.52-43.48)	0.390
GA+AA	GG	52 (39.4)	16 (10.74-21.26)	0.031
GA+AA	GA+AA	31 (23.5)	12 (6.70-17.30)	0.041

**3:**
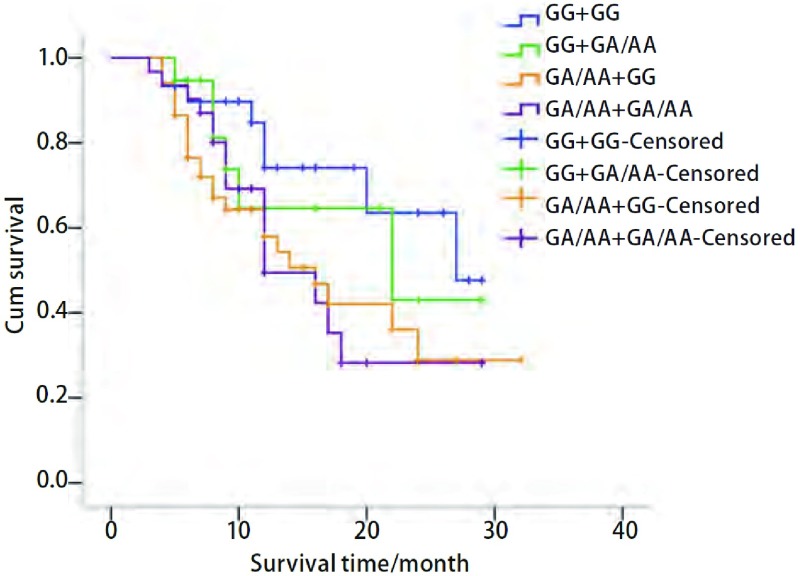
IGF-1R+1013（G/A）和IGF-2R+1619（G/A）联合基因型的生存曲线 *Kaplan*-*Meier* plot of overall survival in relation to the combined genotypes of IGF-1R+1013(G/A) and IGF-2R+1619(G/A)

### 2.3 *Cox*回归模型多因素分析

年龄、性别、吸烟情况、组织类型、临床分期、化疗方案、*IGF*-*1R*和*IGF*-*2R*基因多态性8项变量均进入*Cox*模型进行分析，IGF-1R+1013（G/A）变异等位基因A是影响NSCLC预后的独立危险因素，IGF-1R+1013（G/A）变异等位基因A携带者（GA+AA）的OR是GG基因型携带者的2.104倍（*P*=0.020），IGF-1R+1013（G/A）联合IGF-2R+1619（G/A）基因多态性也是影响NSCLC预后的独立因素（*P*=0.025）。见表 4。

**4:** 晚期NSCLC预后影响因素的*Co*x多因素回归分析结果 *Cox* multivariate regression analysis of prognostic factors in advanced NSCLC patients

Variable	B	SE	Wald	df	*P*	0R
Gender	0.187	0.260	0.269	1	0.604	1.205
Age	0.368	0.294	1.719	1	0.190	1.470
Smoking status	0.618	0.363	2.891	1	0.089	1.854
Histology	-0.202	0.207	0.954	1	0.329	0.817
pTNM stage	0.511	0.299	2.924	1	0.087	1.667
Chemotherapy regimen	0.181	0.123	2.155	1	0.142	1.199
IGF-1R	0.744	0.320	5.403	1	0.020	2.104
IGF-2R	0.012	0.280	0.002	1	0.965	1.012
IGF-1R×IGF-2R^*^	0.289	0.129	5.046	1	0.025	1.335
^*^ Combined polymorphisms of IGF-1R+1013(G/A) and IGF-2R+1619(G/A).						

## 3 讨论

*IGF*-*1R*基因定位于15q25-26，全长约100 kb，有21个外显子，IGF-2R蛋白是一种酪氨酸激酶跨膜蛋白受体，由2个a-亚基（130 kDa-135 kDa）和2个β亚基（90 kDa-95 kDa）通过二硫键结合而形成四聚体（α2β2），与细胞外配体结合后，通过Ras/Raf/MEK/ERK和PI3K/AKT两条信号传导通路起到促进细胞存活及抗细胞凋亡的作用^[7]^。*IGF*-*2R*基因定位于6q26，约136 kb，含有48个外显子，其编码的蛋白质是一个无内在催化活性的跨膜糖蛋白，与IGF-2配体结合后不能传导信号反而加速IGF-2的降解。目前认为IGF-2R对IGF信号转导通路所起的作用很可能是负向调节性的^[8]^。两基因共同维持着细胞生长和凋亡的平衡，而在肿瘤细胞中出现IGF-1R的高表达和/或IGF-2R的低表达促进肿瘤的发生发展并与化疗耐药有关，Lee等^[5]^发现siRNA抑制IGF-1R表达后，顺铂作用A549肺癌细胞所引起的凋亡比例增加；Sangha等^[9]^发现在未经治疗的NSCLC的治疗中，以铂类化疗为基础联合IGF-1R单克隆抗体的治疗方案有较好的效果，尤其是IGF-1R高表达的肺鳞癌患者；某些学者^[10]^还提出同时阻断IGFR及表皮生长因子受体（epidermal growth factor receptor, EGFR）途径较单纯阻断EGFR途径可明显延长肿瘤患者生存期。药理遗传学研究^[11]^表明基因的单核苷酸多态性（single neucleotide polymorphism, SNP）可能导致基因组编码的相应蛋白结构和功能发生改变，从而导致不同个体对疾病的易感性以及对药物的敏感性产生差异。*IGFR*基因存在高度多态性且某些碱基的突变可导致编码蛋白结构和功能的改变，为进一步探讨*IGFR*基因与化疗疗效的关系，本研究选择了与肿瘤相关的IGF-1R+1013（G/A）和IGF-2R+1619（G/A）多态性位点，研究其SNP与化疗疗效及预后的关系。

132例晚期NSCLC患者铂类化疗后的总体有效率（完全缓解+部分缓解）为36.4%，与文献^[12]^报道39.2%接近，未发现*IGF*-*1R*和*IGF*-*2R*基因多态性与铂类化疗的临床受益率有关（*P*＞0.05）。在此基础上以更具有临床意义的生存情况作为指标进一步分析其与晚期肺癌预后的关系，分析发现IGF-1R+1013（G/A）基因多态性单独或联合IGF-2R+1619（G/A）基因多态性与晚期NSCLC生存期有关。Bonafe等^[13]^研究发现IGF-1R+1013位点G→A碱基突变影响血清IGF-1的水平和人类寿命，携带IGF-1R+1013（G/A）突变等位基因A的个体血清IGF-1的水平低、寿命长，由于研究的人群不同，我们发现携带IGF-1R+1013（G/A）突变等位基因A的肺癌患者生存期短，虽然没有检测患者血清中IGF-1的水平，但推测*IGF*-*1R*的基因突变主要通过影响肺癌组织中IGF-1的水平而影响患者的预后，其分子基础是IGF-1R +1013位点编码的谷氨酸，鸟嘌呤（G）→腺嘌呤（A）突变虽然没有导致编码蛋白质的氨基酸改变，但可能通过影响mRNA的转录和稳定性改变增加IGF-1R蛋白的量，促进癌细胞生长分化，加快肺癌的进展，远期疗效表现为生存期缩短。对于IGF-2R+1619（G/A）的基因多态性与肺癌的关系国内外未见报道，本研究首次探讨其基因多态性与晚期肺癌患者化疗疗效的关系，未发现两者之间有相关性，其分子基础可能是*IGF*-*2R*基因+1619位点G→A碱基突变虽然导致其编码的氨基酸改变，但合成的IGF-2R蛋白改变对化疗疗效的影响不明显。

尽管非条件*Logistic*回归分析未能显示IGF-1R+1013（G/A）和IGF-2R+1619（G/A）基因多态性与铂类化疗疗效有关，仍不能排除研究样本量较小、人群种族及个体对药物的特异性不同，同时入组人群给药剂量、给药时间的不同也会对结果造成影响，课题组拟扩大样本继续追踪观察。此外研究发现IGF-1R+1013（G/A）基因多态性单独或联合IGF-2R+1619（G/A）基因多态性与晚期NSCLC生存期有关，然而由于研究样本量不够大，影响化疗预后的因素较多，目前研究所获得的结果只是一种趋势。因此IGF-1R+1013（G/A）和IGF-2R+1619（G/A）的基因多态性与铂类化疗疗效的关系还有待大宗临床研究证实。
